# Probabilistic Spike Propagation for Efficient Hardware Implementation of Spiking Neural Networks

**DOI:** 10.3389/fnins.2021.694402

**Published:** 2021-07-15

**Authors:** Abinand Nallathambi, Sanchari Sen, Anand Raghunathan, Nitin Chandrachoodan

**Affiliations:** ^1^Department of Electrical Engineering, Indian Institute of Technology Madras, Chennai, India; ^2^School of Electrical and Computer Engineering, Purdue University, West Lafayette, IN, United States

**Keywords:** spiking neural networks, hardware acceleration, energy efficiency, memory, probabilistic spike propagation

## Abstract

Spiking neural networks (SNNs) have gained considerable attention in recent years due to their ability to model temporal event streams, be trained using unsupervised learning rules, and be realized on low-power event-driven hardware. Notwithstanding the intrinsic desirable attributes of SNNs, there is a need to further optimize their computational efficiency to enable their deployment in highly resource-constrained systems. The complexity of evaluating an SNN is strongly correlated to the spiking activity in the network, and can be measured in terms of a fundamental unit of computation, *viz*. spike propagation along a synapse from a single source neuron to a single target neuron. We propose *probabilistic spike propagation*, an approach to optimize rate-coded SNNs by interpreting synaptic weights as probabilities, and utilizing these probabilities to regulate spike propagation. The approach results in 2.4–3.69× reduction in spikes propagated, leading to reduced time and energy consumption. We propose Probabilistic Spiking Neural Network Application Processor (P-SNNAP), a specialized SNN accelerator with support for probabilistic spike propagation. Our evaluations across a suite of benchmark SNNs demonstrate that probabilistic spike propagation results in 1.39–2× energy reduction with simultaneous speedups of 1.16–1.62× compared to the traditional model of SNN evaluation.

## 1. Introduction

Spiking Neural Networks (SNNs), often referred to as the third generation of neural networks (Maass, 1997), are attracting a lot of attention due to several desirable characteristics, including their ability to model temporal event streams, the possibility of training them using unsupervised, bio-inspired learning rules such as Spike Timing Dependent Plasticity (STDP) (Bi and Poo, 1998), and the emergence of low-power SNN hardware platforms such as IBM TrueNorth (Akopyan et al., 2015) and Intel Loihi (Davies et al., 2018).

SNNs represent information as discrete spike events and follow an event-driven model of computation, where the work done (and hence, the time or energy consumed) is proportional to the number of spike events. Further, they do not require multiplication to be performed when processing a spike, offering the prospect of reduced hardware complexity compared to conventional Artificial Neural Networks (ANNs). Due to these differences, SNNs are not well-suited to commodity hardware platforms like graphics processing units (GPUs). Further, in contrast to hardware accelerators for ANNs, which usually focus on exploiting regular data parallelism, hardware architectures for spiking networks (e.g., Furber et al., 2014; Neil and Liu, 2014; Akopyan et al., 2015; Roy et al., 2017) focus more on features that enable efficient event-driven computation.

Despite being event driven, spiking networks still require a large number of memory accesses (Neil and Liu, 2014). When a neuron spikes, it is first necessary to identify its fanout neurons, i.e., the connectivity information needs to be fetched along with the weights of the corresponding synapses. Finally, the membrane potentials of the fanout neurons impacted by the spike are fetched and updated. Recent data (Pedram et al., 2017) indicates that fetching data from memory is much more expensive than arithmetic computations. Consequently, developing techniques for reducing the number of memory accesses in SNNs is critical for improving their energy-efficiency.

### 1.1. Probabilistic Spike Propagation

In this paper, we present a probabilistic method of spike propagation that can significantly reduce the number of memory accesses required for the evaluation of a rate-coded spiking neural network, thus saving both run-time and energy. We realize the proposed probabilistic spike propagation mechanism through probabilistic synapses. Conventionally, the weight of a synapse connecting two neurons in an SNN specifies the amount by which the membrane potential of the postsynaptic neuron is increased whenever the presynaptic neuron spikes. Alternatively, inspired by the ideas in Seung (2003) and Kasabov (2010), we could view this weight as a measure of how likely it is that a spike will propagate across the synapse. A probabilistic synapse doesn't propagate all spikes generated by its presynaptic neuron to the postsynaptic neuron. Instead, whenever a neuron spikes, only a subset of its outgoing synapses with weights above a certain randomly-chosen threshold propagate the spike. To minimize the effect of this randomness on a network's accuracy while maximizing the time and energy savings, we develop techniques that generate the random thresholds and perform the synaptic updates in an optimized manner. To summarize, the specific contributions of this work are as follows:

- We propose probabilistic spike propagation, an approach to reduce the cost of spike propagation in rate-coded SNNs. Probabilistic spike propagation reduces the number of memory accesses and consequently reduces the time and energy consumed in evaluating a spiking network.
- We propose techniques that allow probabilistic spike propagation to be applied to existing SNNs and methods to optimize the tradeoff between energy and accuracy degradation.
- We evaluate the approach on a benchmark suite of six SNNs across five image classification datasets and characterize its performance. We also develop P-SNNAP, an SNN accelerator enhanced to support probabilistic spike propagation, on which we evaluate the reductions in energy consumption and run-time.

The paper is organized as follows. First, we present a brief overview of SNN preliminaries in section 2, and motivate the need to optimize spike propagation. In section 3, we discuss the key concepts of probabilistic spike propagation and in section 4, we present the P-SNNAP hardware architecture. We present the results of evaluating the proposed approach in section 6. In section 7, we present related efforts and highlight the unique aspects of our work. Finally, section 8 concludes the paper.

## 2. SNN Preliminaries

**Figure 1:**
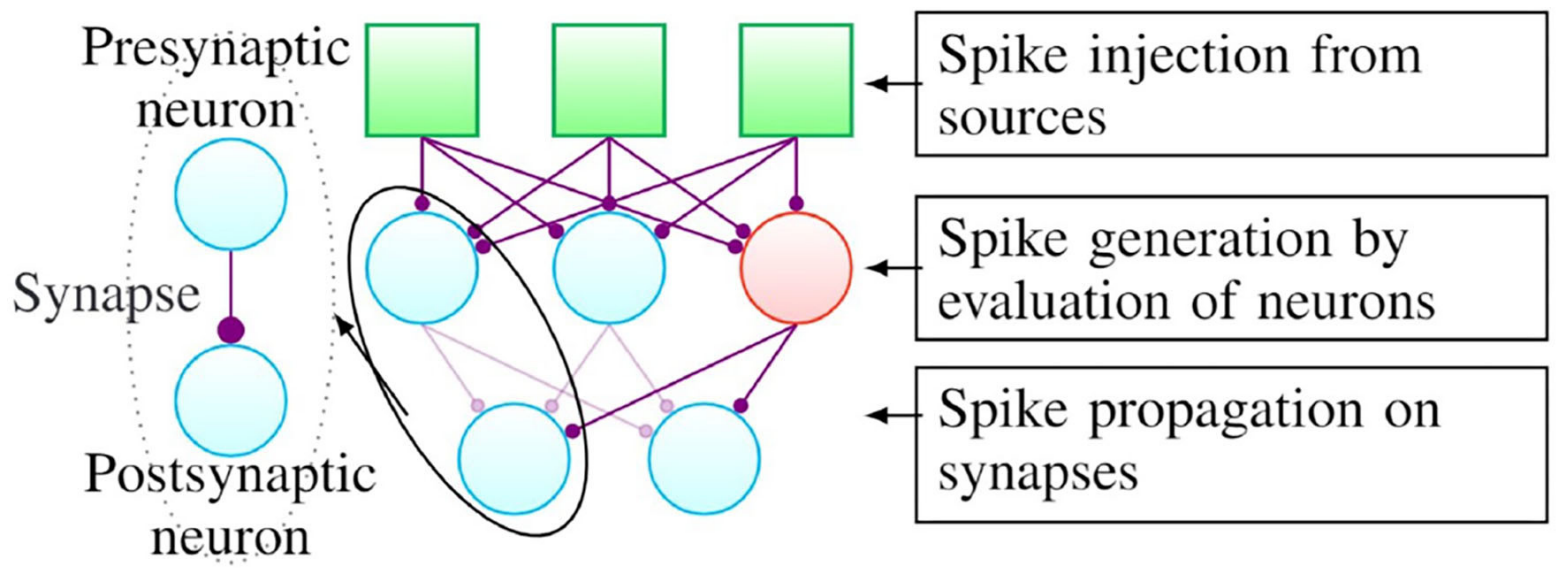
A generic structure of spiking neural networks.

The evaluation of a spiking neural network involves three phases, namely (a) spike injection, (b) spike generation, and (c) spike propagation, as illustrated in Figure 1. Although the illustration is for the case of a simple fully connected network here, the algorithm remains unchanged for arbitrary connectivity patterns. As shown in the figure, the connection between different neurons is referred to as a synapse and the neurons on either side of the connection are referred to as the presynaptic and postsynaptic neuron, respectively.

A more detailed description of SNN evaluation is presented in Algorithm 1. The first phase, spike injection, involves introducing input spikes that initiate activity in the network. These input spikes can be directly obtained from event-based sensors, or can be generated from static inputs through conversion methods. There have been numerous efforts in developing neuromorphic or spiking sensors (Vanarse et al., 2016) and spike based benchmark datasets (Orchard et al., 2015; Hu et al., 2016; Rueckauer and Delbruck, 2016). In many of these efforts, the inputs are presented as Poisson spike trains (Diehl and Cook, 2015) or as analog stimuli directly applied to the membrane potentials of input layer neurons (Rueckauer et al., 2017).

**Algorithm 1:**
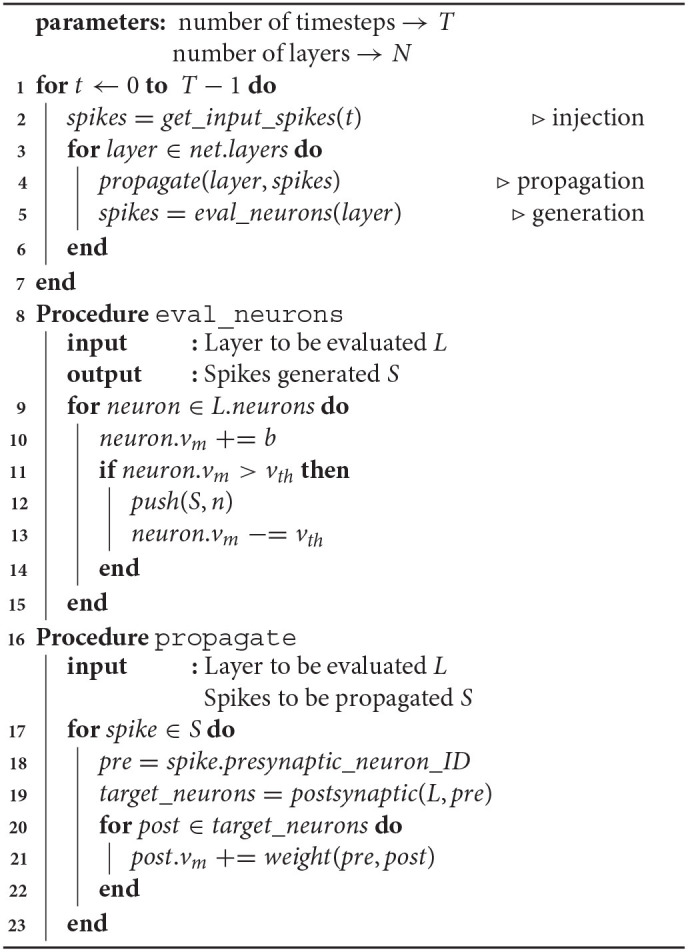
SNN Evaluation Scheme

The second phase, spike generation, is the process of evaluating each neuron and, based on a mathematical model of the neuron, determining whether it produces a spike. Neuron models with varying levels of bio-fidelity have been proposed. In this work, we use the Integrate-and-Fire (IF) neuron model for illustration, but the approach is largely independent of the underlying neuron model. The spike generation step, as described in Algorithm 1 (lines 8–15), typically involves fetching the state variables of the neuron model from memory and performing some arithmetic operations. In the case of the IF model, the membrane potential *v*_*m*_ is fetched and the bias is added to it (line 10). Next, it is checked for firing by comparing it with the threshold voltage *v*_*th*_ (line 11). In case of firing, the neuron ID is pushed into a spike queue (line 12), and the membrane potential is reset and written back to the memory (line 13). If every neuron in the network is evaluated at every timestep, the above process will involve at least one memory access per neuron per timestep. Thus, the number of computations and memory accesses performed during spike generation are proportional to the number of neurons.

The final phase, spike propagation, as described in Algorithm 1 (lines 16–23), is performed in the event of a neuron spiking. For every spike in the queue, the postsynaptic neurons connected to the spiking neuron are identified (line 19) and all such neurons are updated with the respective synaptic weights (line 21). This process is referred to as a synaptic update. It involves fetching the synaptic weight and the state of the postsynaptic neuron from memory, updating the state with the weight, and writing the neuron state back to memory. This amounts to at least two memory reads, one arithmetic operation and one memory write per synapse per spike per timestep. The total number of computations and memory accesses for the propagation step is thus proportional to the amount of spiking activity (number of spikes) and the number of synapses in the network. Overall, as the number of synapses in a network far outnumber neurons, the number of memory accesses associated with the spike propagation step exceeds that of the other two phases and it accounts for the dominant share of memory accesses during SNN valuation.

**Figure 2:**
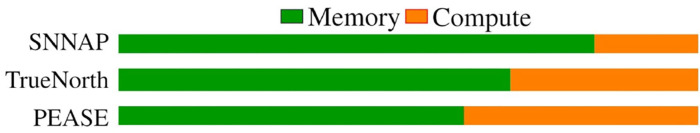
Ratio of memory to compute energy on PEASE, TrueNorth, and SNNAP.

In Figure 2, we show the fraction of energy consumed by memory and compute operations during SNN evaluation on three different hardware platforms, *viz*. IBM TrueNorth (Akopyan et al., 2015), SNNAP (Sen et al., 2017), and PEASE (Roy et al., 2017). It is observed that memory accounts for the predominant portion of energy consumption in each of these hardware platforms. Thus, techniques for improving the energy efficiency of SNNs should focus on reducing memory energy. Further, as discussed above, spike propagation requires more memory accesses than the other phases in SNN evaluation. Hence, to improve energy efficiency of SNN implementations, it is imperative to develop better spike propagation techniques that reduce memory accesses.

## 3. Probabilistic Spike Propagation

We propose a probabilistic approach to spike propagation for reducing the number of memory accesses during SNN evaluation, and consequently the total energy consumed. It can be applied to existing spiking networks while causing minimal-to-zero degradation in recognition accuracy. This section first outlines the key concepts involved and subsequently describes the proposed approach in detail.

### 3.1. Key Concepts

Consider two neurons (labeled *i* and *j*, respectively) in an SNN, that are connected by a synapse. Neuron *i* is called *presynaptic* and neuron *j postsynaptic* if the output of *i* is used to drive the membrane potential of *j*. The magnitude of the weight associated with the synapse is *w*_*ij*_, which we will assume (without loss of generality) to be a real-valued number ∈ [0, 1]. This is a safe assumption since the effects of the weights are evaluated relative to a threshold value, and so it is possible to normalize all weights to this range of magnitudes. Note that negative weights would correspond to *inhibitory* synapses, which are modeled in exactly the same way as *excitatory* synapses, and only the magnitude of the weight matters for the discussion that follows.

The weight associated with the synaptic connection as well as the spiking activity of neuron *i* dictate the amount of impact it has on neuron *j*. We quantify this impact as the total potentiation of a postsynaptic neuron due to a presynaptic neuron. Due to the temporal nature of spiking neural networks, the total potentiation should be measured after incorporating the spikes from neuron *i* across all timesteps. Thus, considering a spiking pattern of *S*_*i*_ for neuron *i* and a synaptic weight of *w*_*ij*_, the total potentiation, *M*_*ij*_, of postsynaptic neuron *j* by neuron *i* at time *t* is

(1) $$\begin{array}{l} M_{ij}^{d}\left(t\right)=C_{i}\left(t\right)\times w_{ij} \end{array}$$

where *C*_*i*_(*t*) is the total number of times neuron *i* has spiked until time *t*. We term this process of spike propagation as deterministic, and denote it using the superscript *d*. It should be noted here that $M_{ij}^{d}\left(t\right)$ is only the impact neuron *i* has on the membrane potential of neuron *j*, while the spiking behavior of neuron *j* itself depends on the neuron model and its potentiation by other presynaptic neurons.

Instead of potentiating neuron *j* by *w*_*ij*_ every time neuron *i* spikes, if we apply a weight of *ŵ*_*ij*_ for a random subset of the spikes, the total potentiation becomes

(2) $$\begin{array}{l} M_{ij}^{p}\left(t\right)={Ĉ}_{i}\left(t\right)\times {ŵ}_{ij} \end{array}$$

*Ŝ*_*i*_(*t*) is the random subset of spikes from neuron *i* that were propagated to neuron *j* and *Ĉ*_*i*_(*t*) is the number of spikes in *Ŝ*_*i*_(*t*) till time *t*. In other words, we propagate a spike from neuron *i* to neuron *j* with a probability of *p*_*ij*_, where

(3) $$\begin{array}{l} p_{ij}=\underset{t\to \infty }{\lim}\frac{{Ĉ}_{i}\left(t\right)}{C_{i}\left(t\right)} \end{array}$$

We term this process of spike propagation as probabilistic, and denote it by the superscript *p*.

We can define the average potentiation of neuron *j* by neuron *i* as follows:

(4) $$\begin{array}{l} \bar{M_{ij}\left(t\right)}=\frac{M_{ij}\left(t\right)}{C_{i}\left(t\right)} \end{array}$$

It should be noted that the average potentiation is defined when there is at least one spike from neuron *i*. For the deterministic approach, the average potentiation is equal to the synaptic weight itself.

(5) $$\begin{array}{ll} \bar{M_{ij}^{d}\left(t\right)} & =w_{ij} \end{array}$$

On the other hand, for the probabilistic approach corresponding to Equation (3), the average potentiation is a limit.

(6) $$\begin{array}{ll} \underset{t\to \infty }{\lim}\bar{M_{ij}^{p}\left(t\right)} & =p_{ij}\times {ŵ}_{ij} \end{array}$$

We hypothesize that, it is sufficient that the average potentiation for probabilistic spike propagation tends toward the average potentiation for the deterministic case, for the network to achieve similar levels of accuracy with both the probabilistic and deterministic approaches. This can be achieved by carefully choosing values for *p*_*ij*_ and *ŵ*_*ij*_ in the probabilistic approach. One interesting choice is to set *p*_*ij*_ = *w*_*ij*_ and *ŵ*_*ij*_ = 1, which is simply an alternative interpretation of each weight as the probability of spike propagation. We highlight the effects of such a probabilistic approach through an example below.

**Figure 3:**
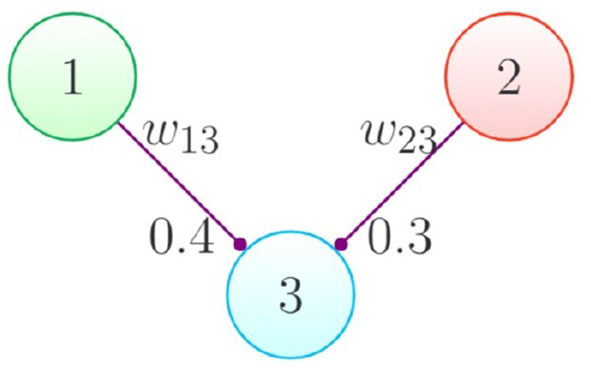
Illustration: Neurons 1 and 2 sending spikes to 3.

**Figure 4:**
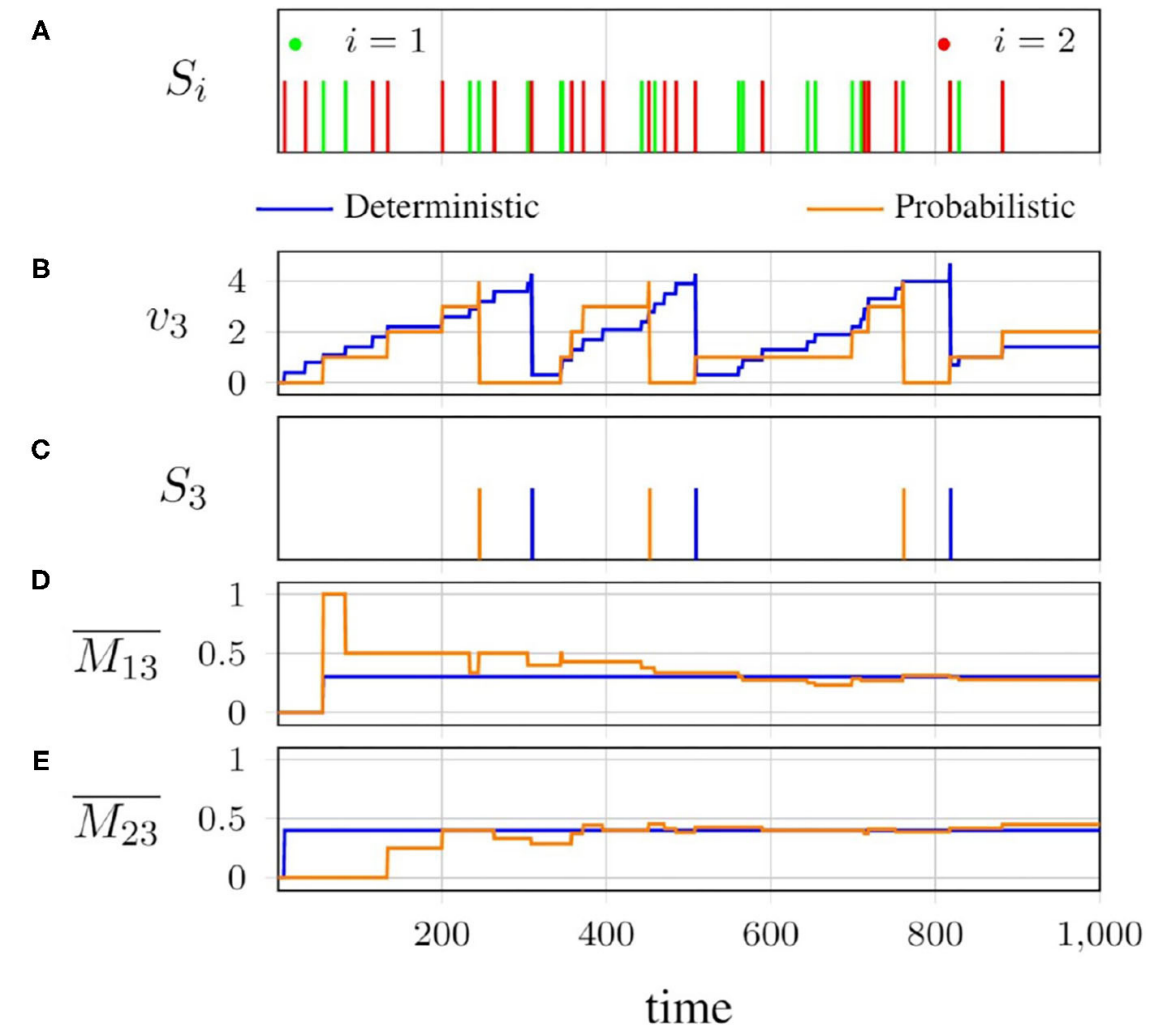
Behavior of the network in Figure 3: **(A)** spike patterns of neurons 1 and 2; **(B)** membrane potential of neuron 3; **(C)** consequent spike pattern of neuron 3; **(D,E)** average potentiation of neuron 3, by neurons 1 and 2, respectively, with deterministic and probabilistic (*p*_*ij*_ = *w*_*ij*_;*ŵ*_*ij*_ = 1) spike propagation.

Consider the connectivity pattern illustrated in Figure 3. Neurons 1 and 2 are spiking sources, which are connected to neuron 3 through synapses. The behavior of this simple network with the deterministic and probabilistic spike propagation approaches is visualized in Figure 4.

The spike patterns of neurons 1 and 2 are shown in Figure 4A. The effects of these spikes on the instantaneous membrane potential of neuron 3 for both the deterministic and probabilistic approaches are shown in Figure 4B. The resulting output spike pattern *S*_3_ for neuron 3 is shown in Figure 4C. As we can see, the probabilistic spike propagation causes the behavior of neuron 3 to slightly differ from that in the deterministic case, but the average potentiations $\bar{M_{13}\left(t\right)}$ and $\bar{M_{23}\left(t\right)}$, which are in shown in Figures 4D,E for the two synapses, respectively show an interesting convergence.

Overall, when the spikes are propagated in a probabilistic fashion, the instantaneous membrane potential of neuron 3 may differ from that under deterministic propagation, but as more and more spikes are generated by the presynaptic neuron, the average potentiation for the probabilistic case converges to the deterministic one, which is essentially the synaptic weight. We introduced randomness into the process of spike propagation in a network that is otherwise deterministic, and allowed the temporal nature of the network to average it out.

The crux of our hypothesis is that even though the introduced randomness alters the instantaneous state of the network, the variations will average out over time and result in a network-level equivalence with the deterministic evaluation scheme. In the following subsections, we describe how to take advantage of this randomness to develop efficient implementations of SNNs.

### 3.2. Accelerating Convergence

From Figure 4, we can infer that, given enough spikes, the average probabilistic potentiation converges to the average deterministic potentiation, which is the synaptic weight.

(7) $$\begin{array}{l} \underset{C_{i}\to \infty }{\lim}\bar{M_{ij}^{p}\left(t\right)}\to w_{ij} \end{array}$$

Clearly, the number of spikes required for convergence is an important issue to address. For better convergence, we would need to process more spikes. The number of spikes is directly related to the number of timesteps for which the network is evaluated. Alternatively, probabilistic spike propagation can be viewed as Monte Carlo sampling for approximating the value of *w*_*ij*_. The number of Bernoulli trials required, which in this case is the number of spikes, for an approximation with low relative error is inversely proportional to the probability of success (Asmussen and Glynn, 2007).

As most weights in neural networks are observed to be small in value, the probabilities of propagation is going to be small for most synapses. And thus, the number of spikes required for convergence of network behavior is going to be large, which directly means that the probabilistic approach will require that the networks be run for more timesteps. Thus, it is desirable to drive up the probabilities of propagation, which will reduce the required number of spikes and consequently bring down the number of timesteps required to converge to the same levels of network performance.

One solution is to let $p_{ij}=\frac{w_{ij}}{w_{i}^{max}}$ and ${ŵ}_{ij}=w_{i}^{max}$, where $w_{i}^{max}$ is the maximum weight of all the outgoing synapses of neuron *i*. For most of the neurons, $w_{i}^{max}$ is lower than 1, which increases the probability of propagation.

**Figure 5:**
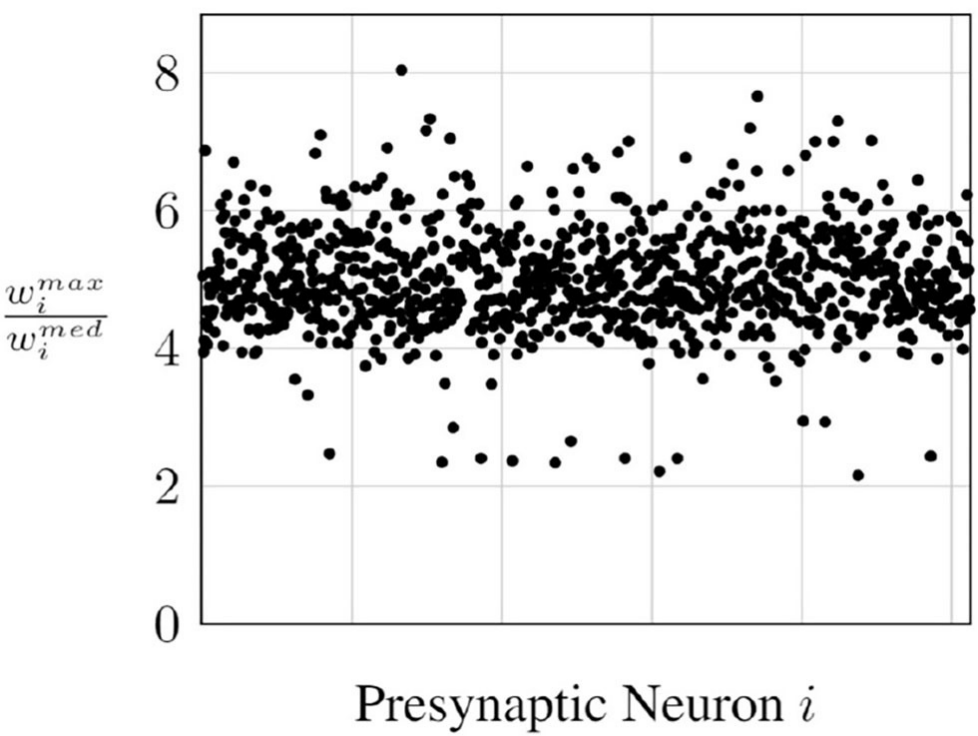
Ratio of maximum to median outgoing weight.

However, the number of outgoing synapses per neuron in modern networks could be in the thousands and, $w_{i}^{max}$, in most cases, tends to be an outlier in the weight distribution. In Figure 5, we see the ratio of $w_{i}^{max}$ to the median outgoing weight $w_{i}^{med}$ for all the neurons in a layer of a fully connected network trained on the MNIST classification task. We observe that $w_{i}^{max}$ is roughly around 5× $w_{i}^{med}$ for most neurons, which means half the outgoing synapses of these neurons in that layer will have spike propagation probabilities of <0.2, which will require a higher number of timesteps for the convergence of the probabilistic approach.

In order to overcome this, we group outgoing synapses of a neuron into synaptic clusters. Synaptic clusters are simply spatial groupings of the outgoing synapses from a neuron. For each synapse, we use the maximum weight of its corresponding cluster as the normalizer, as $p_{ij}=\frac{w_{ij}}{w_{i^{b}}^{max}}$ and ${ŵ}_{ij}=w_{i^{b}}^{max}$, where *b* is the cluster to which the synapse between neurons *i* and *j* belongs. This prevents the synaptic weights from getting dominated by outlier maximum weights, increasing their spike propagation probabilities and accelerating convergence.

**Figure 6:**
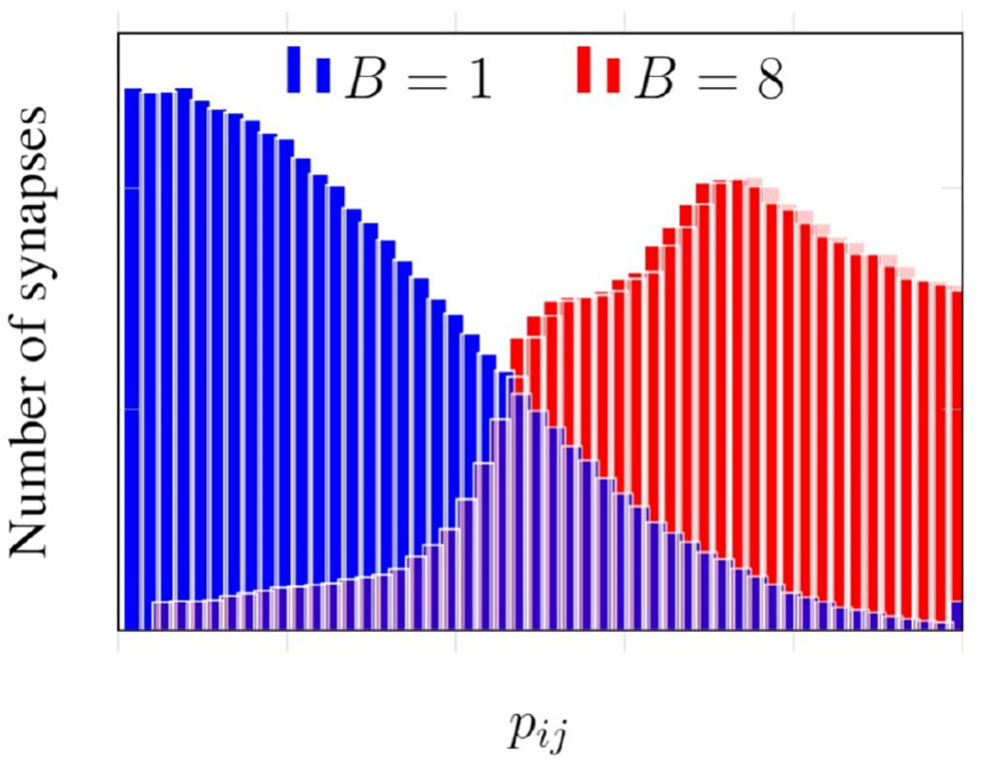
Effect of clustering on probabilities of propagation.

Figure 6 presents histograms for spike propagation probabilities across synapses in the same layer as Figure 5. *B* denotes the number of synaptic clusters in Figure 6. When the outgoing synapses of each neuron are grouped into eight clusters, we see that the histogram is skewed toward higher probabilities, unlike when there is no clustering (*B* = 1).

It is important to note that, for a given number of timesteps, the probability of spike propagation controls a trade-off relationship between cost and performance. The lower the probability, the lower the number of synaptic updates and poorer network performance. The higher the probability, the higher the number of synaptic updates and better network performance. We explore this trade-off in greater detail in section 6.

### 3.3. Realizing the Probabilistic Synapse

A probabilistic synapse can be realized by generating a uniformly distributed random number $r_{b}\in \left[0,w_{i^{b}}^{max}\right]$ and comparing with *w*_*ij*_. The probability of success of this Bernoulli trial is

(8) $$\begin{array}{l} r_{b}\in \left[0,w_{i^{b}}^{max}\right]\to P\left(w_{ij}>r_{b}\right)=\frac{w_{ij}}{w_{i^{b}}^{max}} \end{array}$$

which is equal to the desired probability of propagation presented in the previous discussion. Hence, the spike can be propagated on every synapse that has a weight *w*_*ij*_ greater than *r*_*b*_.

While this implements the probabilistic synapse, it requires fetching of the weight for each synapse from memory prior to the decision of propagation. This can be cheaper than the deterministic approach, as for the synapses that we don't propagate the spikes on, the post-synaptic neurons need not be updated. It should be noted that this random skipping of synapses can cause pipeline inefficiencies in a hardware implementation. In the probabilistic spike propagation process described in Algorithm 2, we overcome this limitation and show how to reduce the memory accesses further. It involves a preprocessing step of organizing synapses into multiple synaptic clusters and sorting the synapses in each cluster by their weights. Along with storing the weights of all the outgoing synapses in sorted order, we also store their indices in rank order (line 5).

**Algorithm 2:**
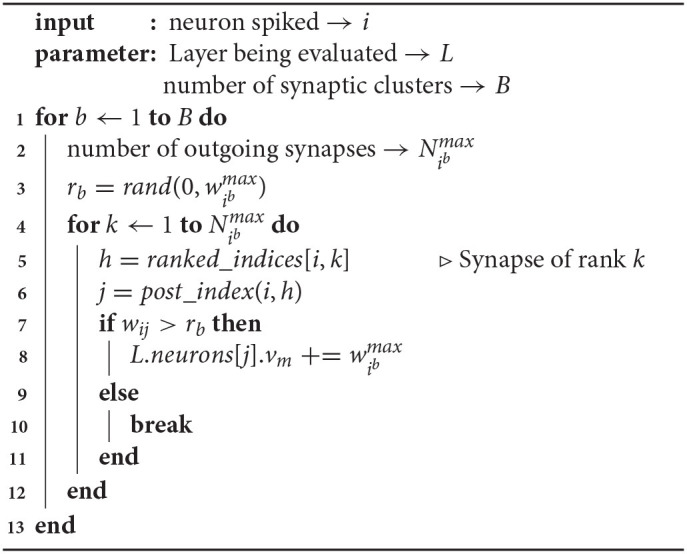
Implementation of Probabilistic Spike Prop.

Consider that neuron *i* has spiked. Assume that the weights of the outgoing synapses of neuron *i* in synaptic cluster *b* are ranked from 1 to $N_{i^{b}}^{max}$ (line 2). As described in Equation (8), for each synaptic cluster, we generate one random number *r*_*b*_ (line 3). Since the weights are stored in sorted order, every synaptic update requires reading the index (line 5) and weight (line 7), but as soon as the comparison fails for one synapse, all the remaining synapses in the cluster can be skipped (line 10). The index *j* of the postsynaptic neuron can be determined with the indices of the presynaptic neuron and the synapse (line 6), based on the underlying connectivity pattern.

#### 3.3.1. Optimization: Determining the Termination Point

We define the *termination point* of the spike propagation from neuron *i*, $t_{p}\in \left[1,N_{i^{b}}^{max}\right]$, as the number of synapses with *w*_*ij*_ > *r*_*b*_. It is the rank of the smallest weight in the synaptic cluster that is greater than *r*_*b*_. In the straightforward method of determining *t*_*p*_ described above, note that we need both the actual weight value and the index of the target neuron, potentially requiring twice the number of memory accesses.

**Figure 7:**
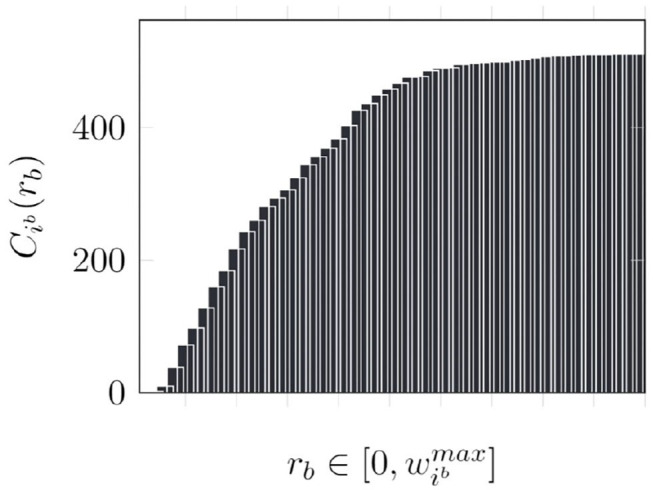
Cumulative histogram of a neuron.

An alternate approach is to use a cumulative histogram of the outgoing weights of each neuron in each cluster, indicated by $C_{i^{b}}$. The cumulative histogram is a discrete function that gives the number of values below the input i.e., $C_{i^{b}}\left(r_{b}\right)$ gives the number of outgoing synapses of neuron *i* in synaptic cluster *b* with weights lesser than *r*_*b*_. Therefore, the termination point *t*_*p*_ is essentially $N_{i^{b}}^{max}-C_{i^{b}}\left(r_{b}\right)$. Thus, by generating and storing a cumulative histogram of the form shown in Figure 7 in a preprocessing step, as shown in Algorithm 3, we can determine *t*_*p*_ through a single memory access (line 4). Consequently, we can perform synaptic updates without fetching the synaptic weights.

**Algorithm 3:**
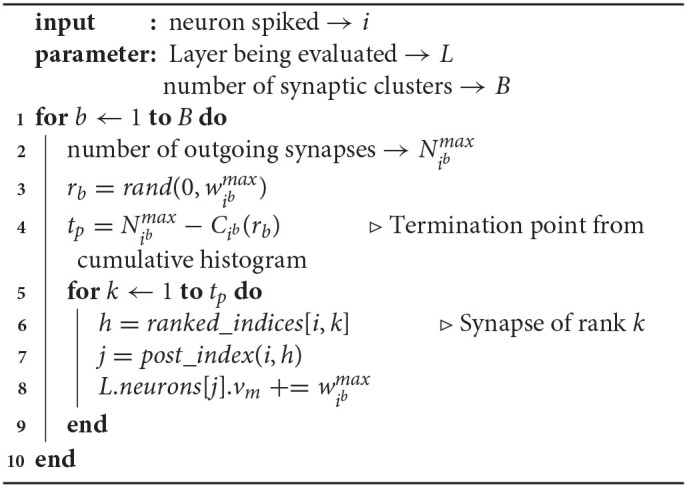
Implementation of Probabilistic Spike Prop.: *t*_*p*_ using Cumulative Histogram

Another way to look at this is as a way of discretizing the space of random number generation for *r*_*b*_. For discrete values of *r*_*b*_, we can store the termination point *t*_*p*_ directly and sample from these points. The number of discrete points correspond to the number of bins of the cumulative histogram and is a parameter of concern. It controls the trade-off between memory overhead and performance. The higher the number of bins, the better the fidelity of the termination point. The lower the resolution, the lower the memory overhead. In this work, we have implemented this approach in the hardware architecture and have studied its implications on accuracy and cost. The trade-off between accuracy and memory overhead has been studied in section 6.

In summary, the proposed probabilistic spike propagation approach reduces the number of synaptic updates, and consequently the number of memory accesses in SNNs by introducing randomness into the process of spike propagation.

## 4. Hardware

**Figure 8:**
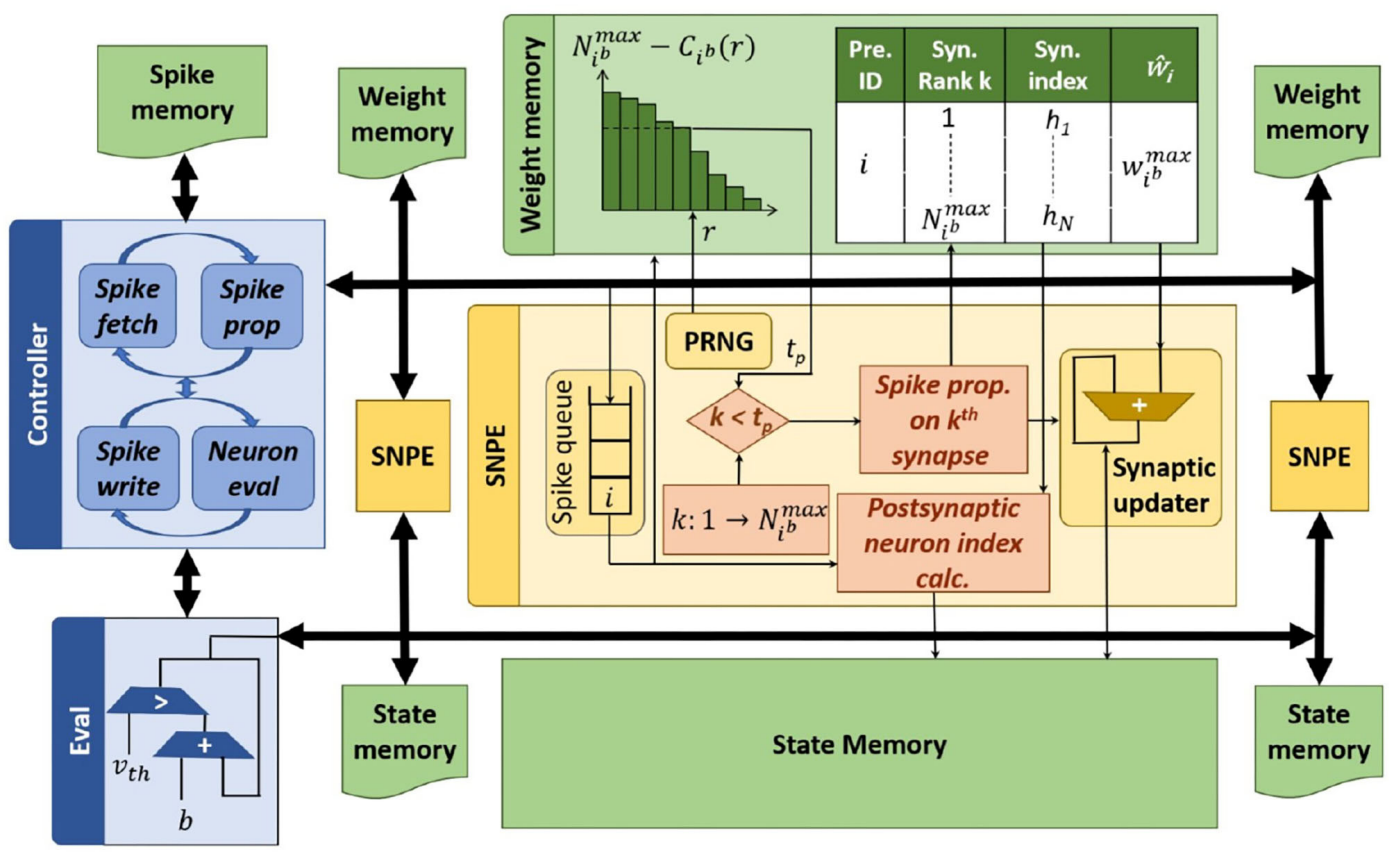
P-SNNAP accelerator architecture.

To evaluate the system-level impact of probabilistic spike propagation, we develop P-SNNAP, an SNN accelerator based on SNNAP (Sen et al., 2017). The overall architecture is shown in Figure 8 and the individual components are described in detail below.

### 4.1. Overview

The P-SNNAP architecture consists of three different modules—the Spike Neural Processing Element (SNPE), the Eval unit and the global controller. It also contains three types of on-chip memories—the spike memory, the weight memory, and the state memory, for storing spikes, weights, and neuronal state variables, respectively.

In a deterministic SNN evaluation, as performed in SNNAP, the neurons in every layer are evaluated at each timestep before moving on to the next timestep. However, it involves loading the neuronal state variables and weights for each layer into the on-chip memory repeatedly at every timestep. To avoid these repeated off-chip memory accesses and increase the reuse of loaded weight values, we evaluate one layer for the total number of timesteps before moving on to the next layer. The spikes generated at each timestep during the evaluation of one layer are stored in the spike memory and subsequently fetched during the evaluation of the next layer. Since a large number of modern deep networks are strictly feed-forward, this layer-wise evaluation scheme can be applied to reduce the required buffering. Specifically, all networks evaluated as part of this work are feed-forward networks. We note that this optimization is orthogonal to our proposal and is applied to both deterministic and probabilistic SNN evaluation.

#### 4.1.1. Eval Unit

The Eval unit, similar to the Leak-and-Spike unit in SNNAP, is the module that performs neuron evaluation. It fetches the membrane potentials from the state memory, increments it by the bias value and compares it with the threshold. If the membrane potential exceeds the threshold, a spike is generated and communicated to the controller. The updated membrane potentials are written back to the state memory.

#### 4.1.2. Controller

The Controller orchestrates the functioning of the accelerator. It has two phases of operation—the first phase controls the SNPEs and the second phase controls the Eval unit. For each timestep, the controller goes through both phases. In the first phase, the controller fetches the spikes generated by the previous layer from the spike memory and sends them to the SNPEs. Once all the spikes are sent and the SNPEs finish their operations, the controller moves on to the second phase, in which the controller receives spikes from the Eval unit as it evaluates all the neurons in the layer and writes the spikes to the spike memory. Once all the neurons of the current layer are evaluated, the current timestep is completed and the controller moves on to the next timestep for the layer.

#### 4.1.3. SNPEs

Spike propagation is realized by an array of Spike Neural Processing Elements (SNPEs). The propagation along different outgoing synapses of neurons are parallelized and balanced across the 16 lanes of the SNPE array. Each lane has an SNPE coupled with two blocks of on-chip memory, one each for membrane potentials and weights. When a layer is evaluated, the controller fetches the spikes from the previous layer and sends them to the SNPEs. On receiving a spike, an SNPE uses the index of the spiking neuron to iterate through its outgoing synpases. For each synapse, the SNPE calculates the index of the post-synaptic neuron. This calculation depends on the connectivity pattern of the layer being evaluated. Next, for each post-synaptic neuron, its membrane potential and the weight of the corresponding synapse are fetched. The membrane potential is updated and written back.

##### 4.1.3.1. Mapping Synaptic Clusters to Lanes

Both the lanes of SNPEs in the architecture and the synaptic clusters in the probabilistic approach group outgoing synapses of neurons. Despite the similarity, the grouping is done with different goals. While deciding the number of lanes, the primary concerns are inference speed and the required logic area and size of the on-chip memories, at the hardware level. On the other hand, while deciding the number of synaptic clusters, the concerns are computational effort and accuracy.

When the number of synaptic clusters and lanes are chosen to be equal, a simple direct mapping is possible—the outer loop in Algorithm 3 is unrolled completely and each SNPE processes one cluster. It is also possible for the number of clusters and lanes to be different. When the number of synaptic clusters is less than the number of lanes, multiple lanes operate on a single synaptic cluster. When the number of synaptic clusters are more than the number of lanes, each lane will have to process more than one cluster, i.e., the outer loop in Algorithm 3 is unrolled partially and each SNPE will process multiple clusters.

The weight memory in each SNPE lane stores all the information required to perform probabilistic spike propagation, including the discretized cumulative histograms for the corresponding mapped synaptic clusters, the sorted synaptic indices and the maximum weight values.

##### 4.1.3.2. Asynchronous Spike Processing

**Figure 9:**
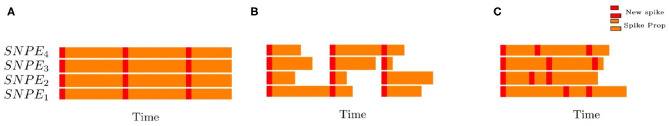
Timing diagrams illustrating the need for asynchronous spike serving for the probabilistic spike propagation. **(A)** Synchronous SNPE-deterministic spike propagation, **(B)** Synchronous SNPE-probabilistic spike propagation, **(C)** Asynchronous SNPE-probabilistic spike propagation.

In the deterministic propagation of spikes, since the outgoing synapses of the spiked neuron are distributed equally among the lanes, each lane ends up performing an equal number of synaptic updates, which means that all the SNPEs take an equal amount of time, as shown in Figure 9A. In contrast, in the probabilistic propagation of spikes, even though the lanes have been assigned an equal number of synapses, the termination point *t*_*p*_ that each lane comes up with is random and thus, they perform different number of synaptic updates and end up taking unequal amounts of time, as illustrated in Figure 9B. Before the controller can serve the next spike, a number of SNPEs would have been idle. These bubbles in the compute pattern in turn leads to under-utilization of SNPEs and compute inefficiencies.

To address the aforementioned challenge, P-SNNAP implements asynchronous spike processing. Each SNPE is equipped with a queue as shown in Figure 8. The controller fills up the queues with spikes. As soon as an SNPE has finished propagating a spike, it can move on to the next spike from the queue, as shown in Figure 9C. This allows the probabilistic approach to be faster and have better compute utilization.

## 5. Experimental Methodology

In this section, we describe the experimental methodology and benchmarks used to evaluate the proposed concepts.

### 5.1. Benchmarks

The benefits of the proposed approach have been studied across a range of image classification networks trained on MNIST, SVHN, CIFAR10, CIFAR100, and ImageNet datasets, as listed in Table 1. The networks were trained as conventional analog (non-spiking) deep networks using backpropagation and converted to spiking networks using the Keras-based ANN-to-SNN conversion and simulation framework developed by Rueckauer et al. (2017).

**Table 1:** Benchmarks.

**Network**	**Neurons**	**Synapses**	**Params**
MNIST-FCN	2 k	1.8 M	1.8 M
MNIST-CNN	112 k	51 M	786 k
SVHN-CNN	130 k	40.7 M	2.3 M
CIFAR10-AllConv	0.2 M	174.9 M	1.4 M
CIFAR100-VGG16	0.3 M	313 M	15 M
ImageNet-VGG16	15 M	15.5 B	138 M

We refer to the deterministic evaluation of all synapses in a network as the baseline (BSL) approach in section 6. On the other hand, for the Probabilistic Spike Propagation (PSP) approach in section 6, we empirically choose between deterministic or probabilistic spike propagation at a layer-granularity for each network in the benchmark suite, with the goal of iso-timesteps operation. The probabilistic approach is beneficial only for layers with large numbers of synaptic connections and high activity. For instance, the CIFAR10-AllConv network in our benchmark suite is the All-CNN-C network from Springenberg et al. (2014), that was converted into a spiking network. In PSP, layers 2, 4, 5, and 7 of this network were evaluated with probabilistic spike propagation, while the remaining layers were evaluated with deterministic spike propagation. The savings achieved by this configuration are reported in section 6.

### 5.2. P-SNNAP Details

The P-SNNAP engine was designed at the Register Transfer Level and synthesized to the Nangate 15 nm technology using Synopsys Design Compiler. CACTI (Thoziyoor et al., 2008) was used to model the memory blocks. A simulator was implemented in the dynamic, high level language Julia (Bezanson et al., 2017) to simulate the proposed spike propagation methods on P-SNNAP. The hardware simulator profiles the memory accesses and number of cycles and uses the values obtained from hardware synthesis and CACTI to estimate energy consumption. The compute logic in P-SNNAP occupies a total area of 0.1 *mm*^2^. The compute power consumption is 28.6 *mW*. A version of SNNAP without support for probabilistic spike propagation was implemented to act as the baseline in our comparisons. We observe that the probabilistic approach incurs a compute logic area overhead of 12% and compute logic power overhead of 23.5%. These hardware additions facilitate significant improvements in time and energy consumed to evaluate SNNs, as discussed in the following section.

In our implementation, the on-chip memory in the accelerator was sufficient to hold the largest layer in the suite of benchmarks. The on-chip memory can be reduced if needed by employing the layer-wise evaluation scheme at a finer granularity and dividing layers into multiple blocks of neurons and evaluating one block at a time. The memory overhead of the probabilistic approach is due to the tables of cumulative histograms and these tables are sparsely accessed at the rate of 1 read per lane per spike. Hence, these cumulative histogram tables can reside in off-chip DRAM and fetched on demand if on-chip memory is constrained.

## 6. Results

In this section, we present results of our experiments that evaluate the benefits of probabilistic spike propagation (PSP) in SNNs.

### 6.1. Accuracy vs. Synaptic Updates

**Figure 10:**
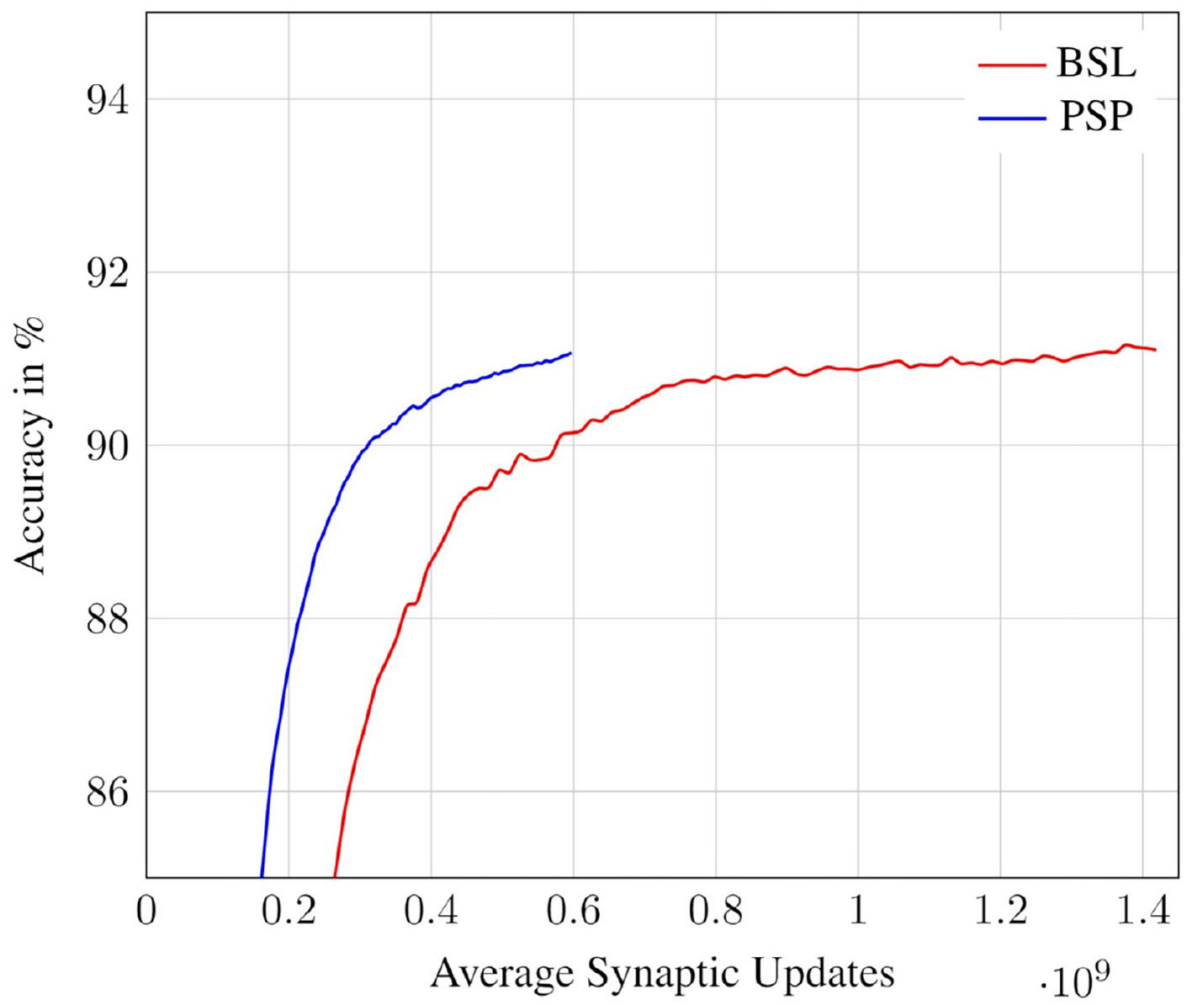
Accuracy vs. Synaptic updates: PSP performance in the CIFAR10-AllConv network.

**Figure 11:**
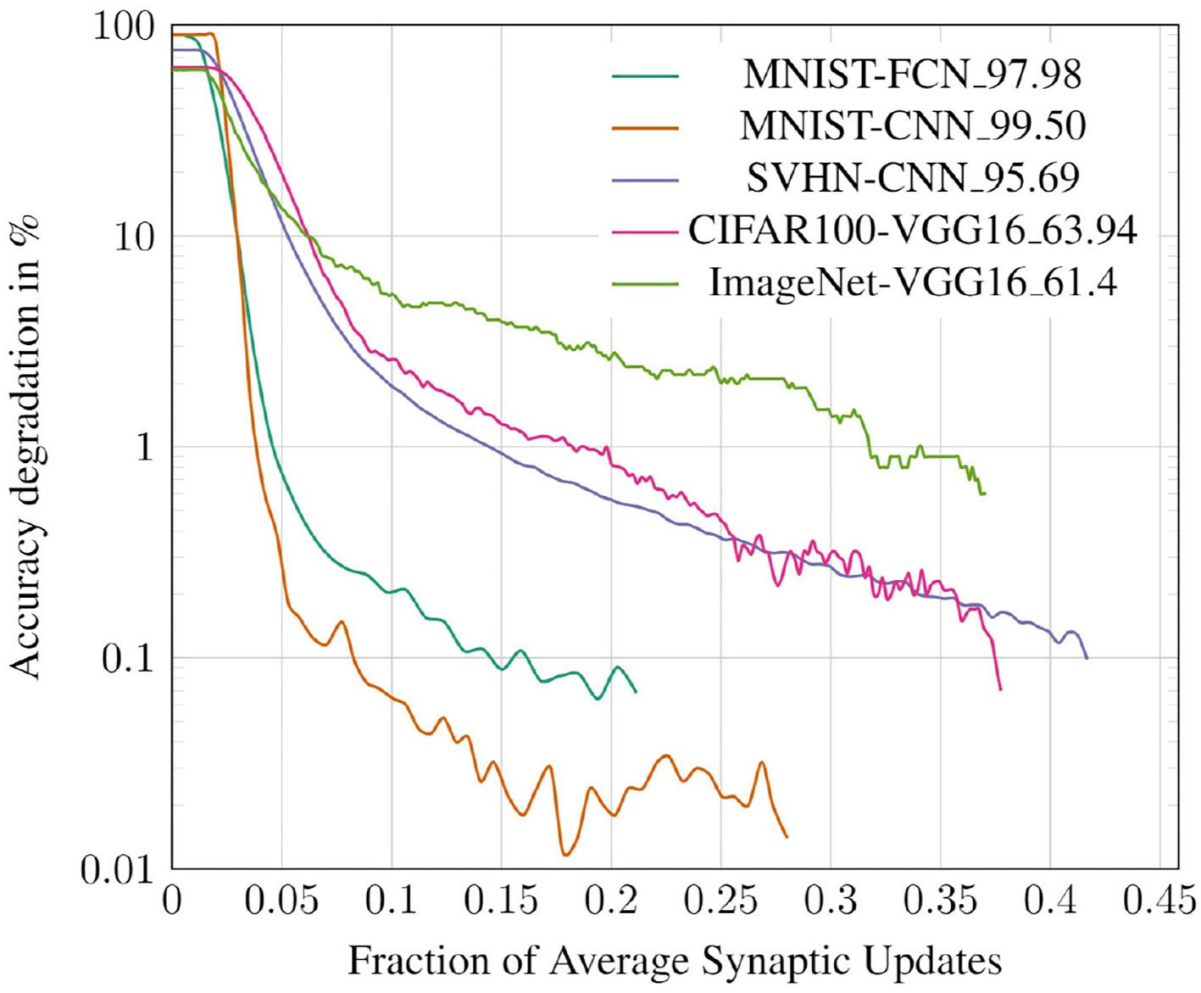
Accuracy degradation vs. synaptic updates for various benchmarks.

In this subsection, we study the trade-off between classification accuracy of a network and the number of synaptic updates performed during its evaluation. Specifically, we record the classification accuracy and number of synaptic updates (averaged across all test inputs) at each timestep for both the deterministic and probabilistic propagation schemes. The results for the CIFAR10 all-convolutional network are presented in Figure 10. We observe that, for both approaches, accuracy saturates with increasing timesteps, and hence with increasing synaptic updates. We also observe that the proposed probabilistic approach requires significantly fewer synaptic updates than the baseline to achieve roughly iso-accuracy. In Figure 11, we visualize the accuracy degradation of PSP as a function of synaptic updates (normalized to a fraction of the baseline) for the other networks in the benchmark suite. The accuracy degradation and synaptic update fraction were calculated with respect to the respective final values of BSL. The final accuracy values of the BSL networks are noted in the legend. PSP causes very minimal accuracy degradations of <0.1% in the networks trained on the MNIST, SVHN, CIFAR10, and CIFAR100 tasks. The ImageNet-VGG16 network was evaluated on subset of 1,000 images of the ImageNet validation set and an accuracy degradation of 0.6% was observed.

### 6.2. Reductions in Synaptic Updates, Energy, and Run-Time

**Figure 12:**
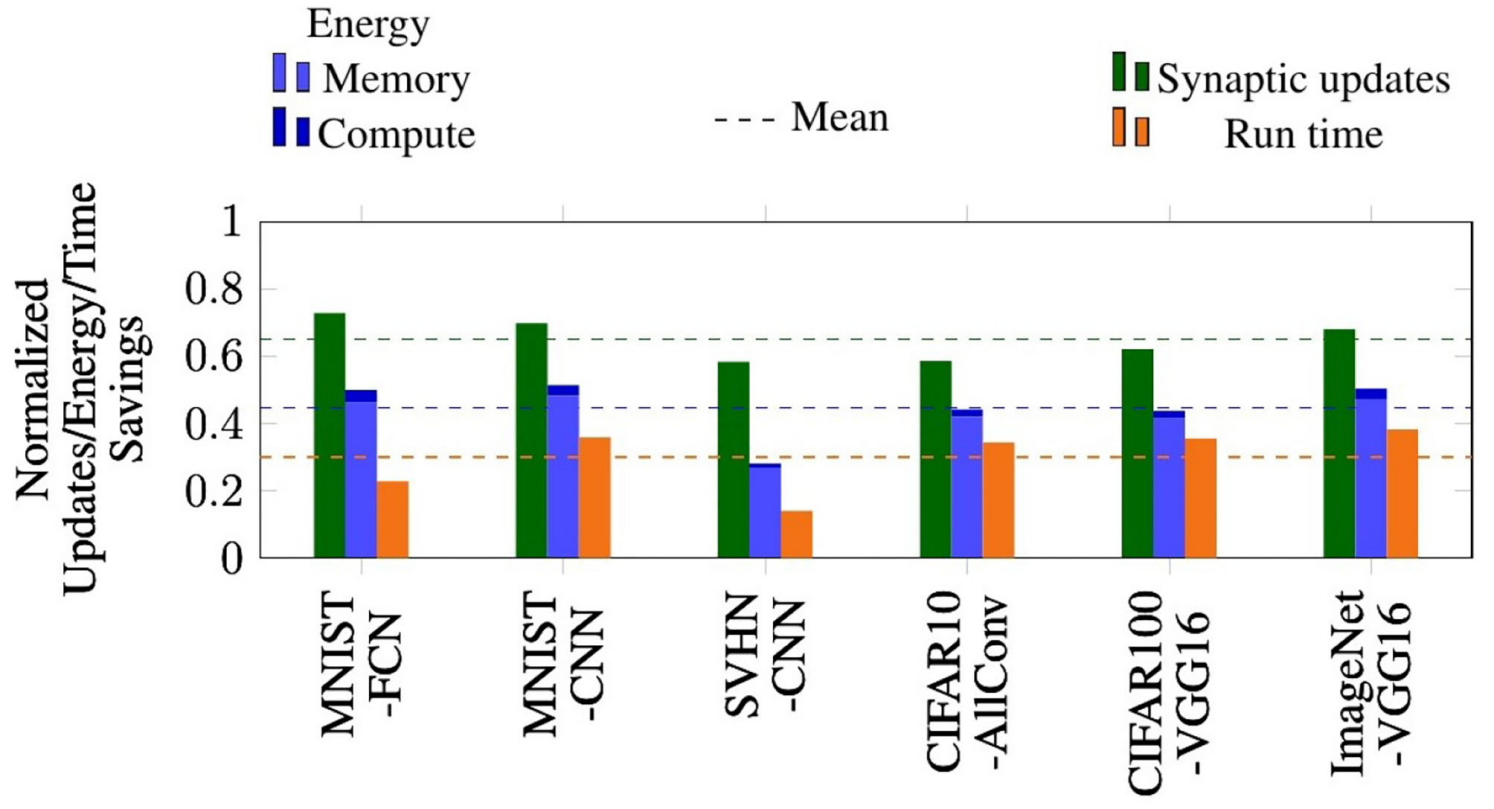
Performance benefits of probabilistic spike propagation on P-SNNAP.

The benefits of PSP in terms of the reduction in the number of synaptic updates, total energy, and execution time on the P-SNNAP architecture are presented in Figure 12. The BSL and PSP cases were evaluated for iso-timesteps and the corresponding number of synaptic updates, energy and execution time were measured. We observe that PSP achieves 2.4–3.69× reduction in average number of synaptic updates per inference across all benchmarks. It should be noted that the reduction in synaptic updates for a specific network depends on the distribution of weights, which is why there is some variability across the benchmark suite. These benefits translate to 1.39–2× reduction in average total energy per inference. Clearly, the bulk of the energy benefits can be attributed to the reduction in memory accesses. As a result of the asynchronous spike serving, the probabilistic spike propagation approach also achieves a 1.16–1.62× speedup on the P-SNNAP architecture.

### 6.3. Number of Synaptic Clusters vs. Accuracy

As discussed in section 3.2, increasing the number of synaptic clusters causes the number of synapses affected by outlier weights to go down, and their probabilities of propagation go up.

We observe that this improves the classification accuracy for iso-synaptic updates. In the extreme case, with 1 synapse per cluster, probabilistic propagation becomes identical to the deterministic approach. This dictates that the trade-off relationship between number of synaptic updates and accuracy has a sweet spot on the possible number of synaptic clusters.

**Figure 13:**
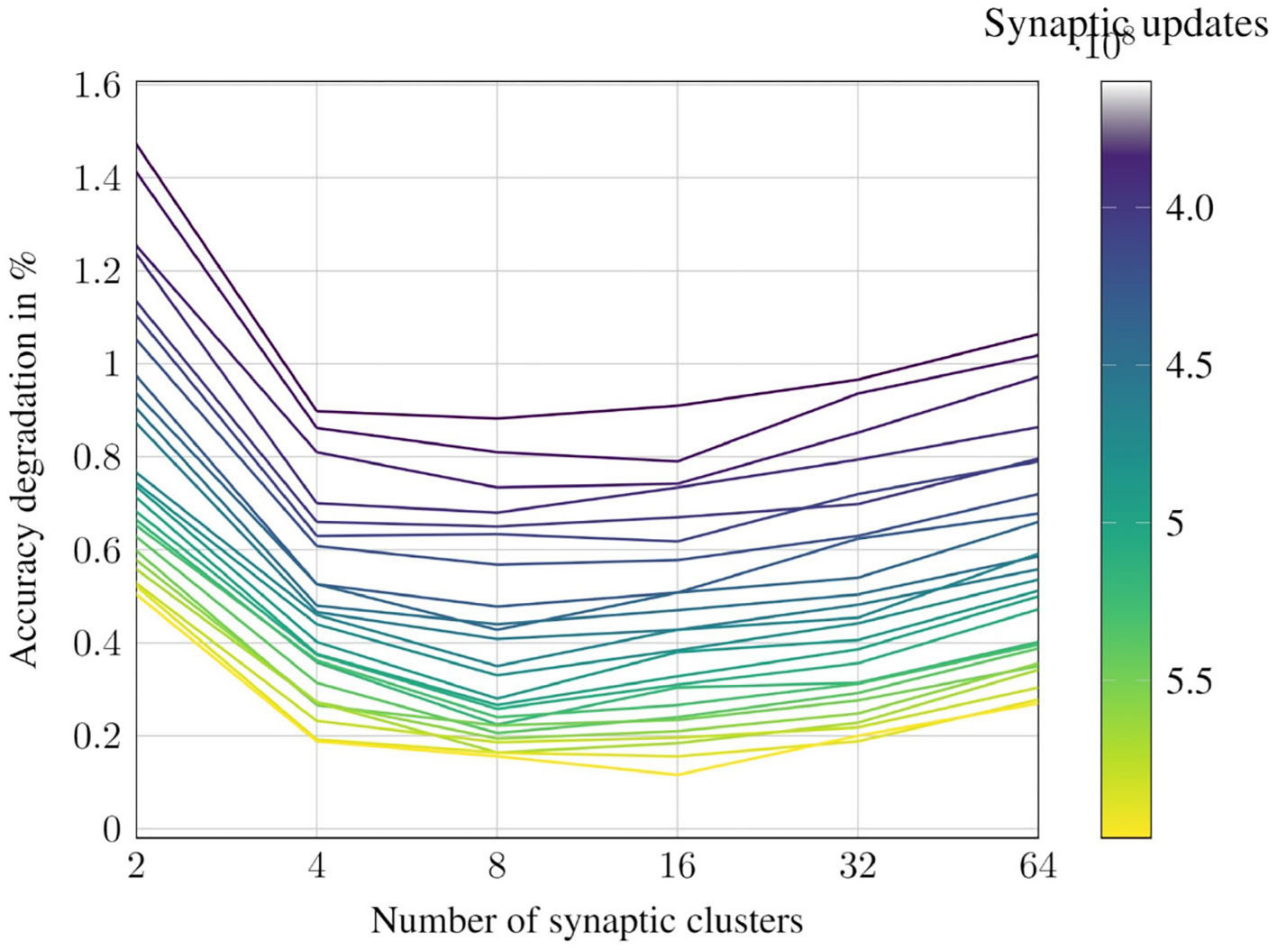
Impact of varying the number of synaptic clusters for CIFAR10-AllConv.

The all-convolutional CIFAR10 network has been studied to explore this relationship in more detail. The network is evaluated with different number of synaptic clusters and the accuracy and average number of synaptic updates per inference image are measured. The contour plot in Figure 13 visualizes this surface. Each line in the contour represents the accuracy degradation for different number of synaptic clusters at a particular level of computational effort, or, number of synaptic updates. We observe that across our benchmark suite, the most favorable trade-off is achieved when the number of synaptic clusters is set to 8 or 16.

It should be noted that this sweet spot is dependent, at a high level, on the number of synapses per cluster, which is decided by the size of the network. Ideally, the number of synaptic clusters could be determined at a per-neuron granularity. However, in this work, we have chosen it to be a network-level hyperparameter to reduce the overall search space.

### 6.4. Resolution of the Cumulative Histogram

The number of bins used in the cumulative histogram impacts the fidelity of the random number *r*_*b*_, as it affects the value of the termination point *t*_*p*_ determined from the cumulative histogram. Therefore, it directly affects the degradation in classification accuracy. At the same time, reducing the number of bins reduces the memory footprint. It should be noted that, the number of accesses to determine *t*_*p*_ is only one per lane per spike, irrelevant of the number of bins used in the cumulative histogram.

**Figure 14:**
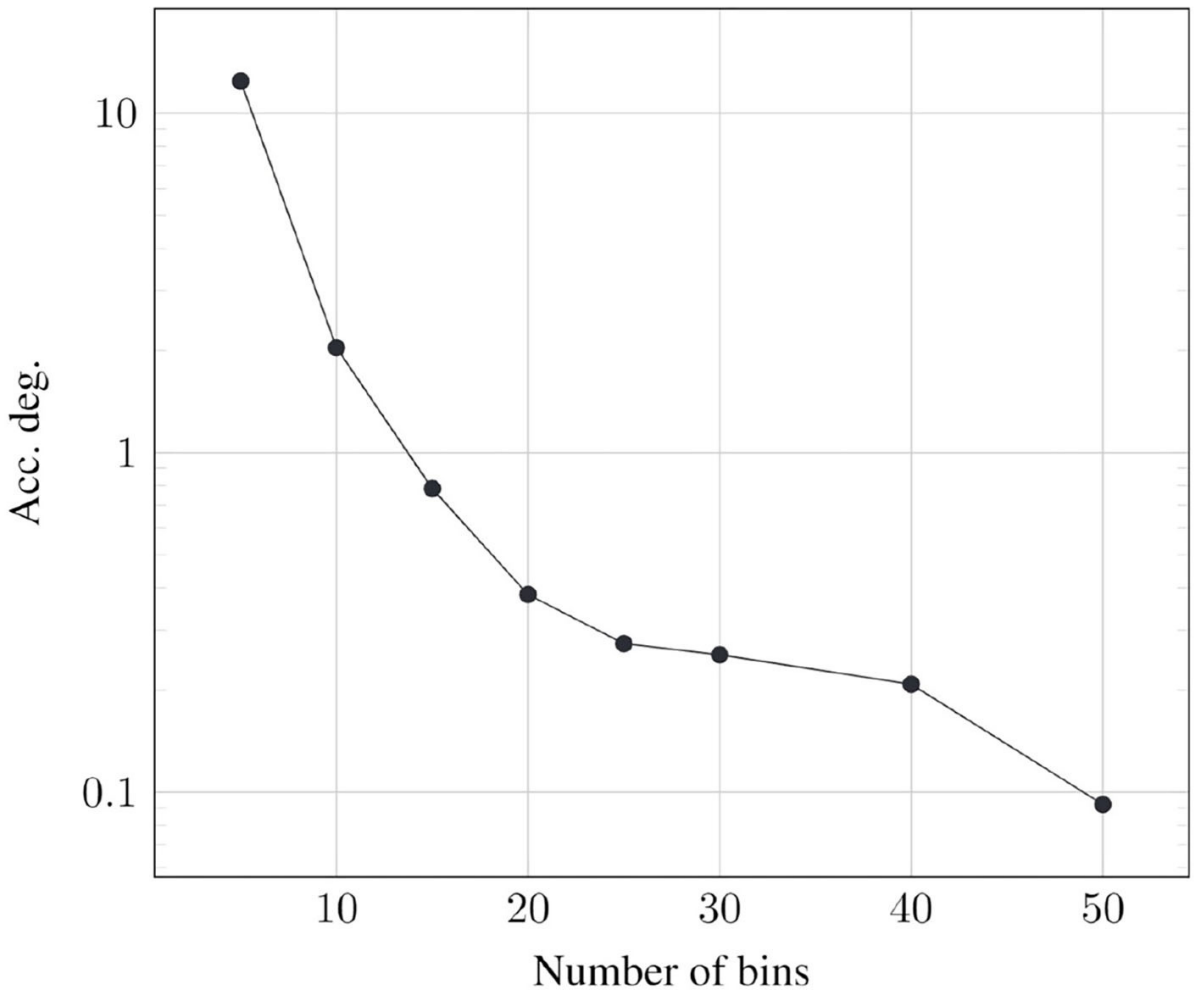
Optimal resolution of cumulative histogram for CIFAR10-AllConv.

We specifically study the effect of the number of bins on the classification accuracy of CIFAR10 all-convolutional network. Figure 14 plots the corresponding degradation in recognition accuracy as a function of the number of bins. As expected, we observe that the accuracy degradation reduces as the number of bins is increased.

A cumulative histogram of 50 bins causes a memory overhead of 23.7% in the CIFAR10-AllConv network. While this can be considered to be significant, we note the following

- The memory overhead is much lower in larger models like CIFAR100-VGG16 (10.9%) and ImageNet-VGG16 (1.1%).
- Although the memory footprint is larger, the total number of memory accesses with PSP is substantially lower.

## 7. Related Works

The focus of this work is to improve the energy efficiency of spiking neural networks by utilizing a probabilistic approach to spike propagation for reducing the number of memory accesses. It can be directly applied to pre-trained spiking networks, without any structural or behavioral modifications. We now relate this to previously proposed approaches for improving SNN implementations and highlight the unique aspects of our approach.

### 7.1. Custom Hardware Architectures

There have been several custom hardware accelerators designed expressly to implement spiking networks (Neil and Liu, 2014; Akopyan et al., 2015; Cheung et al., 2016; Smaragdos et al., 2017; Davies et al., 2018). They employ specialized compute and communication units to match the computational and communication pattern in SNNs. Our approach is complementary to such techniques, and can potentially be realized on these hardware architectures with some memory overheads.

### 7.2. Stochastic Techniques

Stochastic computation techniques apply randomness to the process of computation itself (Shanbhag et al., 2010). Variants of this approach have been applied to spiking neural networks (Rosselló et al., 2012; Ahmed et al., 2016; Smithson et al., 2016). These are mostly orthogonal to the ideas we discuss, since a different (stochastic) hardware architecture for elementary compute units can also be incorporated into our approach which introduces randomness in the process of spike propagation.

### 7.3. Specialized Neuron Models and Encoding Schemes

Ahmed et al. (2016) considered a probabilistic model of the neuron itself, wherein the spike generation mechanism is stochastic in nature but spike propagation is deterministic. Bayesian spiking neurons (Deneve, 2008; Paulin and Van Schaik, 2014) apply probabilistic techniques for the neuron models to perform Bayesian inference. The idea of interpreting synaptic weights as probabilities of spike propagation has also been explored in previous efforts (Seung, 2003; Kasabov, 2010; Neftci et al., 2016). However, these works are primarily algorithmic efforts focused on developing new functionality or new training schemes and don't leverage the randomness to improve energy efficiency. We, on the other hand, demonstrate how randomness can be introduced in the spike propagation of existing spiking networks without changing their intrinsic spiking behavior, while exploiting their time averaging capabilities. We further develop techniques to leverage this randomness for improving the energy efficiency of SNNs. Park et al. (2019) demonstrated neural information coding schemes that improve the energy efficiency of SNN evaluation. This is orthogonal to the direction our work, which improves energy efficiency of existing rate coding networks.

### 7.4. Pruning and Approximate Computing

Pruning is a technique used to reduce memory footprint of neural networks. Rathi et al. (2018) propose a pruning algorithm that works in parallel with STDP SNN learning algorithm on shallow networks. Kundu et al. (2021) propose a pruning algorithm that compresses an ANN during training, converts the network into an SNN, and then retrains the network using a surrogate-gradient based supervised sparse learning. These works prune the networks statically and result in a sparse network model. While these sparse networks can be very lightweight, they lack memory regularity. Developing hardware implementation for these sparse and irregular networks is a niche of its own. Probabilistic spike propagation can be viewed as a stochastic online pruning scheme. Without requiring any retraining, or losing memory regularity, probabilistic spike propagation is able to leverage temporality of SNNs and dynamically reduce memory accesses.

Approximate computing is well-known in the area of signal processing and neural network hardware, but has seen limited application to spiking networks. One example is Sen et al. (2017), where neurons are progressively trimmed from evaluation as time progresses. Another is Krithivasan et al. (2019), where spike propagations are reduced by dynamically bundling spike events across time. Our approach is parallel to these, and could possibly be combined to further reduce computations.

### 7.5. Emerging Technologies

Finally, there are approaches that rely on the use of new and emerging technologies, such as spin-based computing (Sengupta et al., 2016; Zhang et al., 2016; Srinivasan et al., 2017; Chen et al., 2018; Sahu et al., 2018), photonics (De Lima et al., 2017; Chakraborty et al., 2019; Xiang et al., 2019), and memristors (Afifi et al., 2009; Serrano-Gotarredona et al., 2013; Al-Shedivat et al., 2015). These works develop hardware implementations leveraging intrinsic characteristics of these technologies to exhibit properties of spiking networks like leakage, stochasticity, or learning. While this work is focused on the contemporary generation of CMOS computing, our approaches should be applicable to emerging computing technologies.

## 8. Conclusions

In this work, we introduce probabilistic spike propagation as a new approach for improving the energy efficiency of spiking neural networks. The proposed approach reduces the number of memory accesses during the spike propagation phase in SNNs by casting spike propagation as a probabilistic process. We show that the temporal nature of SNNs allows the network to regain any accuracy loss caused by this approach. We successfully apply the technique on pre-trained spiking networks without any network modifications or retraining and demonstrate significant reductions in the number of synaptic updates performed during evaluation while maintaining near iso-accuracy performance levels. We further develop a new hardware architecture, P-SNNAP, to realize probabilistic spike propagation in hardware and show that the proposed approach achieves considerable execution time and energy savings when compared to deterministic spike propagation.

## Data Availability Statement

Publicly available datasets were analyzed in this study. This data can be found at: http://yann.lecun.com/exdb/mnist/; http://ufldl.stanford.edu/housenumbers/; https://www.cs.toronto.edu/~kriz/cifar.html; http://www.image-net.org/.

## Author Contributions

AN implemented the experimental framework. All authors contributed to the conception of the ideas, design of the experiments, analysis of the results, and development of the manuscript.

## Conflict of Interest

SS was employed at IBM Thomas J. Watson Research Center. The remaining authors declare that the research was conducted in the absence of any commercial or financial relationships that could be construed as a potential conflict of interest.
